# Crystal structures of ethyl (2S*,2′R*)-1′-methyl-2′′,3-dioxo-2,3-di­hydro­dispiro­[1-benzo­thio­phene-2,3′-pyrrolidine-2′,3′′-indoline]-4′-carboxyl­ate and ethyl (2S*,2′R*)-5′′-chloro-1′-methyl-2′′,3-dioxo-2,3-di­hydro­dispiro­[1-benzo­thio­phene-2,3′-pyrrolidine-2′,3′′-indoline]-4′-carboxyl­ate

**DOI:** 10.1107/S1600536814015426

**Published:** 2014-07-19

**Authors:** M. P. Savithri, M. Suresh, R. Raghunathan, G. Vimala, R. Raja, A. SubbiahPandi

**Affiliations:** a Department of Physics, Queen Mary’s College (Autonomous), Chennai 600 004, India; b Department of Organic Chemistry, University of Madras, Guindy Campus, Chennai 600 025, India; c Department of Physics, Presidency College (Autonomous), Chennai 600 005, India

**Keywords:** di­spiro, pyrrolidine-indole, benzo­thio­phene, crystal structure

## Abstract

In the title compounds, C_22_H_20_N_2_O_4_S, (I), and C_22_H_19_ClN_2_O_4_S, (II), the pyrrolidine rings have twist conformations on the spiro–spiro C—C bonds. In (I), the five-membered ring of the oxindole moiety has an envelope conformation with the spiro C atom as the flap, while in (II) this ring is flat (r.m.s. deviation = 0.042 Å). The mean planes of the pyrrolidine rings are inclined to the mean planes of the indole units [r.m.s deviations = 0.073 and 0.069 Å for (I) and (II), respectively] and the benzo­thio­phene ring systems (r.m.s. deviations = 0.019 and 0.034 Å for (I) and (II), respectively) by 79.57 (8) and 88.61 (7)° for (I), and by 81.99 (10) and 88.79 (10)° for (II). In both compounds, the eth­oxy­carbonyl group occupies an equatorial position with an extended conformation. The overall conformation of the two mol­ecules differs in the angle of inclination of the indole unit with respect to the benzo­thio­phene ring system, with a dihedral angle between the planes of 71.59 (5) in (I) and 82.27 (7)° in (II). In the crystal of (I), mol­ecules are linked *via* pairs of N—H⋯O hydrogen bonds, forming inversion dimers enclosing *R*_2_^2^(14) loops. The dimers are linked *via* C—H⋯O and bifurcated C—H⋯O(O) hydrogen bonds, forming sheets lying parallel to (100). In the crystal of (II), mol­ecules are again linked *via* pairs of N—H⋯O hydrogen bonds, forming inversion dimers but enclosing smaller *R*_2_^2^(8) loops. Here, the dimers are linked by C—H⋯O hydrogen bonds, forming ribbons propagating along [010].

## Abstract 2

The title compounds, (I) and (II), are di­spiro-indole-pyrrolidine-benzo­thio­phene derivatives, with (II) having a chlorine substituent on the oxo­indole unit. As a result, the conformation of the two mol­ecules differs in the angle of inclination of the indole moiety with respect to the benzo­thio­phene ring system, with a dihedral angle of 71.59 (5) in (I) and 82.27 (7)° in (II).

## Chemical context   

The spiro-indole-pyrrolidine ring system is a frequently encountered structural motif in many biologically important and pharmacologically relevant alkaloids, such as vincrinstine, vinblastine and spiro­typostatins (Cordell, 1981 ▸). Highly functionalized pyrrolidines have gained much inter­est in the past few years as they constitute the main structural element of many natural and synthetic pharmacologically active compounds (Waldmann, 1995 ▸). Optically active pyrrolidines have been used as inter­mediates, chiral ligands or auxiliaries in controlled asymmetric synthesis (Suzuki *et al.*, 1994 ▸; Huryn *et al.*, 1991 ▸). In view of this importance, the title compounds were synthesized and we report herein on their mol­ecular and crystal structures.
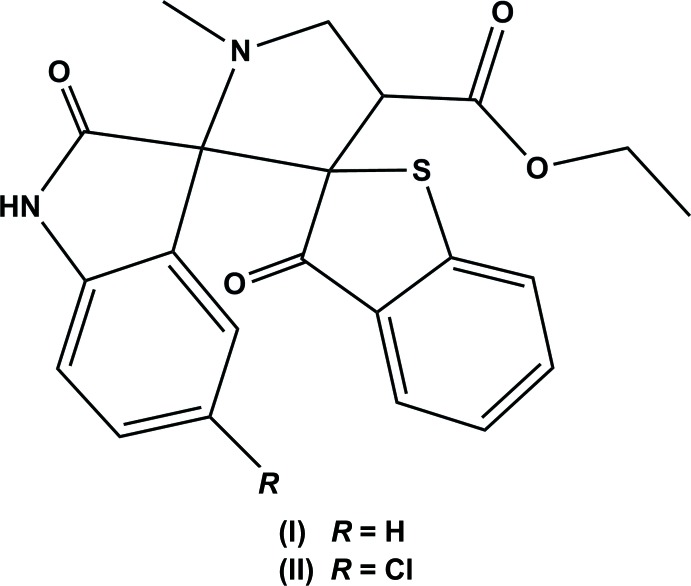


## Structural commentary   

**Figure 1:**
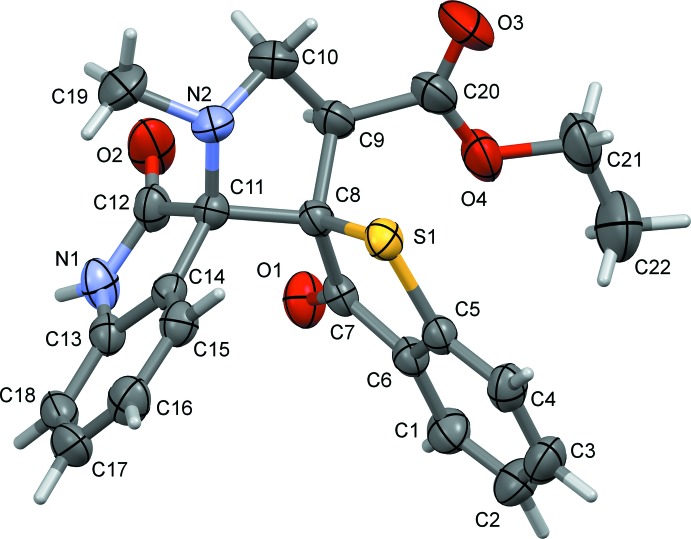
The mol­ecular structure of mol­ecule (I), with the atom labelling. Displacement ellipsoids are drawn at the 50% probability level.

The mol­ecular structure of mol­ecule (I) is shown in Fig. 1 ▸. The pyrrolidine ring (N2/C8–C11) exhibits a twist conformation on bond C8—C11. The five-membered ring (N1/C11–C14) of the oxindole moiety adopts an envelope conformation with C11 as the flap atom. The C12=O2 bond length of 1.213 (1) Å confirms the presence of a keto group in the indoline moiety. The benzo­thio­phene ring system (S1/C1–C8; r.m.s. deviation = 0.019 Å) and the mean plane of the indole ring system (N1/C11–C18; r.m.s. deviation = 0.073 Å) are inclined to one another by 71.59 (5)°, and are both almost normal to the mean plane of the pyrrolidine ring (N2/C8–C11) with dihedral angles of 88.61 (17) and 79.57 (8)°, respectively.

**Figure 2:**
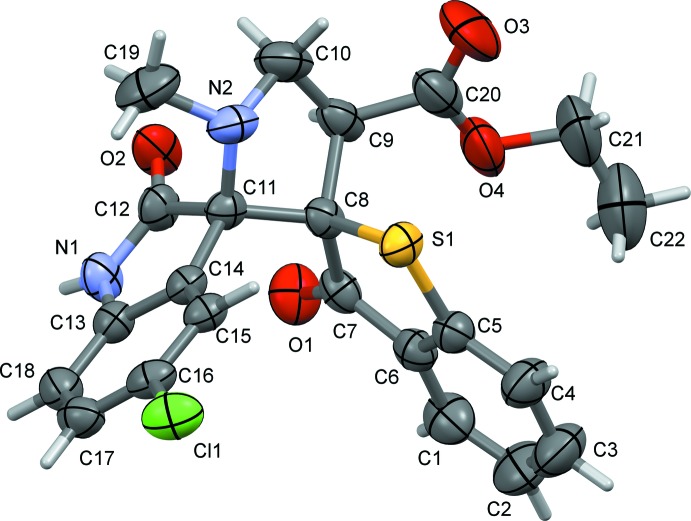
The mol­ecular structure of mol­ecule (II), with the atom labelling. Displacement ellipsoids are drawn at the 50% probability level.

The mol­ecular structure of the compound (II) is illustrated in Fig. 2 ▸. The overall geometry of the mol­ecule is similar to that of (II). The pyrrolidine ring (N2/C8–C11) also adopts a twist conformation on the C8—C11 bond, and the five-membered ring (N1/C11–C14) of the oxindole moiety has an r.m.s. deviation = 0.042 Å. The mean plane of the benzo­thio­phene ring system (S1/C1–C8; r.m.s. deviation = 0.034 Å) and the mean plane of the indole ring system (N1/C11–C18; r.m.s. deviation = 0.069 Å) are inclined to one another by 82.27 (7)°, and are both almost normal to the mean plane of the pyrrol­idine ring (N2/C8–C11) with dihedral angles of 88.79 (10) and 81.99 (10)°, respectively.

**Figure 3:**
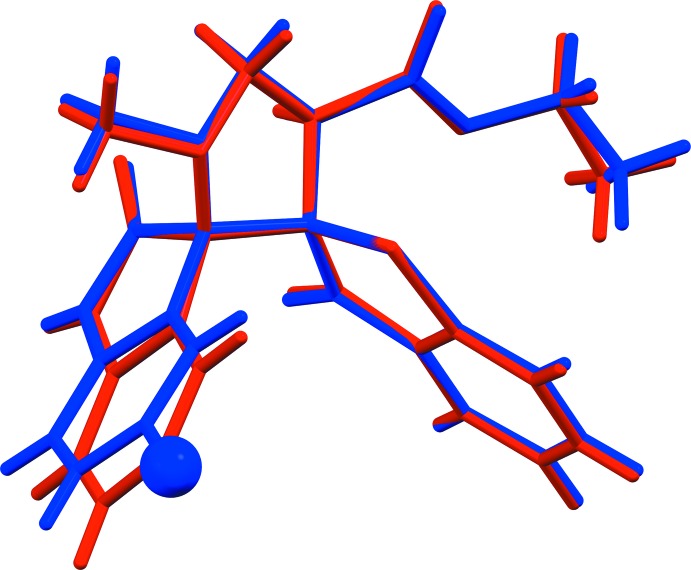
A view of the mol­ecular superposition of mol­ecules (I) and (II) [red (I); blue (II); Cl atom in (II) is shown as a blue ball (*Mercury*; Macrae *et al.*, 2008 ▸)].

Mol­ecules (I) and (II) differ only in the presence of a chloride atom at position 5 in the oxo­indole unit in (II). The conformation of the two mol­ecules differ in the angle of inclination of the indole moiety with respect to the benzo­thio­phene ring system, with a dihedral angle of 71.59 (5) in (I) and 82.27 (7)° in (II). This is illustrated in Fig. 3 ▸, which shows a view of the superposition of the two mol­ecules (*Mercury*; Macrae *et al.*, 2008 ▸). There is also a small difference in the orientation of the ester function, the C20—O4—C21—C22 torsion angle being 173.44 (19) in (I) and 162.3 (3)° in (II).

## Supra­molecular features   

**Figure 4:**
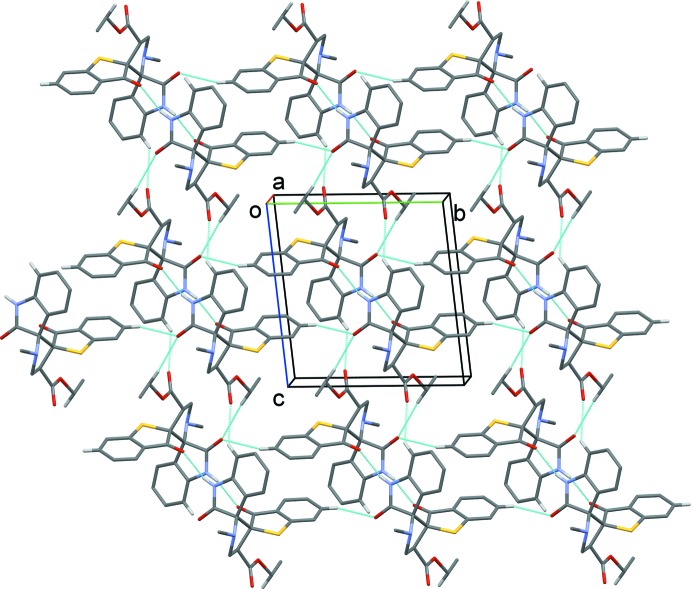
The crystal packing of compound (I), viewed along the *a* axis. The hydrogen bonds are shown as dashed lines (see Table 1 ▸ for details; H atoms not involved in hydrogen bonding have been omitted for clarity).

In the crystal of (I), mol­ecules are linked *via* pairs of N—H⋯O hydrogen bonds, forming inversion dimers enclosing 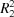
(14) loops (Table 1 ▸ and Fig. 4 ▸). The dimers are linked *via* C—H⋯O and bifurcated C—H⋯O(O) hydrogen bonds, forming sheets lying parallel to (100).

**Figure 5:**
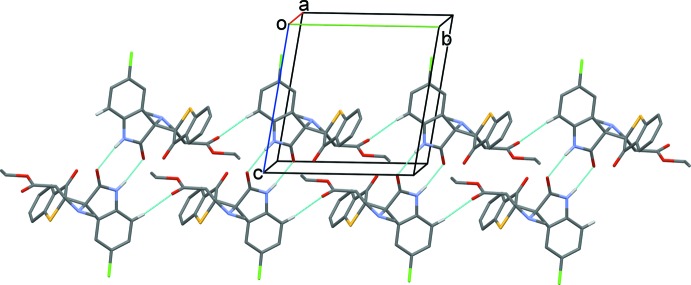
A partial view along the *a* axis of the crystal packing of compound (II). The hydrogen bonds are shown as dashed lines (see Table 2 ▸ for details; H atoms not involved in hydrogen bonding have been omitted for clarity).

In the crystal of (II), mol­ecules are again linked *via* pairs of N—-H⋯O hydrogen bonds, forming inversion dimers but enclosing smaller 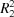
(8) loops (Table 2 ▸ and Fig. 5 ▸). Here the dimers are linked by C—H⋯O hydrogen bonds, forming double-stranded chains propagating along [010].

## Database survey   

A search of the Cambridge Structural Database (Version 5.35, last update November 2013; Allen, 2002 ▸) revealed that the title compounds are the first examples of di­spiro-indole-pyrrolidine derivatives with a benzo­thio­phene substituent on the pyrrolidine ring creating the second spiro C atom. There are a large number of indole-spiro-pyrrolidine compounds but there was only one hit for a di­spiro-indole-pyrrolidine-‘cyclo­pentane-type’ compound, namely 4′-(*p*-meth­oxy­phenyl)-1′-methyl-1*H*-indole-3-spiro-2′-pyrrolidine-3′-spiro-1′′-cyclo­pentane-2(3*H*),2′′-dione (refcode: ILIMUL; Govind *et al.*, 2003 ▸). The geometry of the pyrrolidine and oxindole ring systems of the two mol­ecules compare well with those reported for similar structures, for example, ethyl 1′′-benzyl-2′′-oxo-2′,3′,5′,6′,7′,7a′-hexa­hydro-1′*H*-di­spiro­[indeno[1,2-*b*]-quinoxaline-11,2′-pyrrolizine-3′,3′′-indoline]-1′-carboxyl­ate monohydrate (refcode: IFOVUW; Kannan *et al.*, 2013*a* ▸) and methyl 5′′-chloro-1′,1′′-dimethyl-2,2′′-dioxodi­spiro­[indo­line-3,2′-pyrrolidine-3′,3′′-indoline]-4′-carboxyl­ate (refcode: IFOQUR; Kannan *et al.*, 2013*b* ▸).

## Synthesis and crystallization   

The two compounds were prepared in a similar manner using isatin (1.1 mmol) for (I) and 5-chloro isatin (1.1 mmol) for (II). A mixture of (*E*)-ethyl 2-(3-oxobenzo[*b*]thio­phen-2(3*H*)-yl­idene) acetate (1.0 mmol) and the relevant isatin together with sarcosine (1.1 mmol) was refluxed in methanol (20 ml) until completion of the reaction, as evidenced by TLC analysis. After completion of the reaction, the solvent was evaporated under reduced pressure. The crude reaction mixture was dissolved in di­chloro­methane (2 × 50 ml) and washed with water followed by brine solution. The organic layer was separated and dried over sodium sulfate. After filtration, the solvent was evaporation under reduced pressure. The product was separated by column chromatography using hexane and ethyl acetate (9:1) as eluent to give a white solid. This was dissolved in chloro­form (3 ml) and heated for 2 min. The resulting solutions were allowed to evaporate slowly at room temperature and yielded colourless block-like crystals of compounds (I) and (II).

## Refinement   

Crystal data, data collection and structure refinement details are summarized in Table 3 ▸. For both mol­ecules (I) and (II), the NH H atoms were located in difference Fourier maps and freely refined. The C-bound H atoms were included in calculated positions and treated as riding atoms: C—H = 0.93–0.98 Å with *U*_iso_(H) = 1.5*U*_eq_(C-meth­yl) and = 1.2*U*_eq_(C) for other H atoms.

## Supplementary Material

Crystal structure: contains datablock(s) global, I, II. DOI: 10.1107/S1600536814015426/su2728sup1.cif

Structure factors: contains datablock(s) I. DOI: 10.1107/S1600536814015426/su2728Isup2.hkl

Structure factors: contains datablock(s) II. DOI: 10.1107/S1600536814015426/su2728IIsup3.hkl

CCDC references: 1011623, 1011624

Additional supporting information:  crystallographic information; 3D view; checkCIF report

## Figures and Tables

**Table 1:** Hydrogen-bond geometry (Å, °) for (I) *Cg* is the centroid of the C1–C6 ring.

*D*—H⋯*A*	*D*—H	H⋯*A*	*D*⋯*A*	*D*—H⋯*A*
N1—H1*N*⋯O1^i^	0.83 (2)	2.09 (2)	2.890 (2)	164 (2)
C3—H3⋯O2^ii^	0.93	2.56	3.385 (2)	148
C18—H18⋯O3^iii^	0.93	2.56	3.299 (2)	136
C21—H21*B*⋯O2^iv^	0.97	2.59	3.560 (2)	174
C2—H2⋯*Cg* ^v^	0.93	2.81	3.649 (2)	151

Symmetry codes: (i) 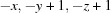; (ii) 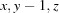; (iii) 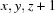; (iv) 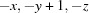; (v) 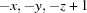.

**Table 2:** Hydrogen-bond geometry (Å, °) for (II) *Cg* is the centroid of the C1–C6 ring.

*D*—H⋯*A*	*D*—H	H⋯*A*	*D*⋯*A*	*D*—H⋯*A*
N1—H1*N*⋯O2^i^	0.81 (2)	2.03 (2)	2.842 (2)	172 (2)
C18—H18⋯O3^ii^	0.93	2.57	3.496 (3)	171
C2—H2⋯*Cg* ^iii^	0.93	2.83	3.649 (2)	155

Symmetry codes: (i) 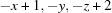; (ii) 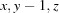; (iii) 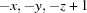.

**Table 3:** Experimental details

	(I)	(II)
Crystal data		
Chemical formula	C_22_H_20_N_2_O_4_S	C_22_H_19_ClN_2_O_4_S
*M*_r_	408.46	442.90
Crystal system, space group	Triclinic, *P* 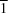	Triclinic, *P* 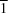
Temperature (K)	293	293
*a*, *b*, *c* (Å)	8.7196 (4), 10.7874 (5), 11.3488 (5)	10.4678 (5), 10.9074 (5), 11.5652 (5)
α, β, γ (°)	82.624 (2), 82.775 (2), 79.214 (2)	85.973 (2), 65.612 (2), 62.089 (2)
*V* (Å^3^)	1034.27 (8)	1050.26 (9)
*Z*	2	2
Radiation type	Mo *K*α	Mo *K*α
μ (mm^−1^)	0.19	0.31
Crystal size (mm)	0.35 × 0.30 × 0.30	0.35 × 0.30 × 0.30

Data collection		
Diffractometer	Bruker Kappa APEXII CCD	Bruker AXS kappa *APEX2* CCD
Absorption correction	Multi-scan (*SADABS*; Sheldrick, 1996 ▸)	Multi-scan (*SADABS*; Sheldrick, 1996 ▸)
*T*_min_, *T*_max_	0.938, 0.946	0.931, 0.940
No. of measured, independent and observed [*I* > 2σ(*I*)] reflections	18982, 3745, 3389	16876, 3788, 3178
*R*_int_	0.024	0.024
(sin θ/λ)_max_ (Å^−1^)	0.600	0.600

Refinement		
*R*[*F* ^2^ > 2σ(*F* ^2^)], *wR*(*F* ^2^), *S*	0.032, 0.087, 1.05	0.036, 0.103, 1.11
No. of reflections	3745	3788
No. of parameters	268	276
H-atom treatment	H atoms treated by a mixture of independent and constrained refinement	H atoms treated by a mixture of independent and constrained refinement
Δρ_max_, Δρ_min_ (e Å^−3^)	0.25, −0.17	0.29, −0.27

Computer programs: *APEX2*, *SAINT* and *XPREP* (Bruker, 2004 ▸), *SHELXS97* and *SHELXL2013* (Sheldrick, 2008 ▸), *Mercury* (Macrae *et al.*, 2008 ▸), *PLATON* (Spek, 2009 ▸), and *publCIF* (Westrip, 2010 ▸).

## supplementary crystallographic information

### (I) Ethyl (2S*,2'R*)-1'-methyl-2'',3-dioxo-2,3-dihydrodispiro[1-benzothiophene-2,3'-pyrrolidine-2',3''-indoline]-4'-carboxylate Crystal data

**Table:**

C_22_H_20_N_2_O_4_S	*V* = 1034.27 (8) Å^3^
*M**_r_* = 408.46	*Z* = 2
Triclinic, *P*1	*F*(000) = 428
Hall symbol: -P 1	*D*_x_ = 1.312 Mg m^−^^3^
*a* = 8.7196 (4) Å	Mo *K*α radiation, λ = 0.71073 Å
*b* = 10.7874 (5) Å	θ = 2.4–25.0°
*c* = 11.3488 (5) Å	µ = 0.19 mm^−^^1^
α = 82.624 (2)°	*T* = 293 K
β = 82.775 (2)°	Block, colourless
γ = 79.214 (2)°	0.35 × 0.30 × 0.30 mm

### (I) Ethyl (2S*,2'R*)-1'-methyl-2'',3-dioxo-2,3-dihydrodispiro[1-benzothiophene-2,3'-pyrrolidine-2',3''-indoline]-4'-carboxylate Data collection

**Table:**

Bruker Kappa APEXII CCD diffractometer	3745 independent reflections
Radiation source: fine-focus sealed tube	3389 reflections with *I* > 2σ(*I*)
Graphite monochromator	*R*_int_ = 0.024
ω and φ scans	θ_max_ = 25.3°, θ_min_ = 2.4°
Absorption correction: multi-scan (*SADABS*; Sheldrick, 1996)	*h* = −10→10
*T*_min_ = 0.938, *T*_max_ = 0.946	*k* = −12→12
18982 measured reflections	*l* = −13→13

### (I) Ethyl (2S*,2'R*)-1'-methyl-2'',3-dioxo-2,3-dihydrodispiro[1-benzothiophene-2,3'-pyrrolidine-2',3''-indoline]-4'-carboxylate Refinement

**Table:**

Refinement on *F*^2^	Secondary atom site location: difference Fourier map
Least-squares matrix: full	Hydrogen site location: mixed
*R*[*F*^2^ > 2σ(*F*^2^)] = 0.032	H atoms treated by a mixture of independent and constrained refinement
*wR*(*F*^2^) = 0.087	*w* = 1/[σ^2^(*F*_o_^2^) + (0.0417*P*)^2^ + 0.2979*P*] where *P* = (*F*_o_^2^ + 2*F*_c_^2^)/3
*S* = 1.05	(Δ/σ)_max_ < 0.001
3745 reflections	Δρ_max_ = 0.25 e Å^−^^3^
268 parameters	Δρ_min_ = −0.17 e Å^−^^3^
0 restraints	Extinction correction: *SHELXL2013* (Sheldrick, 2008), Fc^*^=kFc[1+0.001xFc^2^λ^3^/sin(2θ)]^-1/4^
Primary atom site location: structure-invariant direct methods	Extinction coefficient: 0.0099 (17)

### (I) Ethyl (2S*,2'R*)-1'-methyl-2'',3-dioxo-2,3-dihydrodispiro[1-benzothiophene-2,3'-pyrrolidine-2',3''-indoline]-4'-carboxylate Special details

**Table:**

Geometry. All esds (except the esd in the dihedral angle between two l.s. planes) are estimated using the full covariance matrix. The cell esds are taken into account individually in the estimation of esds in distances, angles and torsion angles; correlations between esds in cell parameters are only used when they are defined by crystal symmetry. An approximate (isotropic) treatment of cell esds is used for estimating esds involving l.s. planes.

### (I) Ethyl (2S*,2'R*)-1'-methyl-2'',3-dioxo-2,3-dihydrodispiro[1-benzothiophene-2,3'-pyrrolidine-2',3''-indoline]-4'-carboxylate Fractional atomic coordinates and isotropic or equivalent isotropic displacement parameters (Å^2^)

**Table:**

	*x*	*y*	*z*	*U*_iso_*/*U*_eq_	
S1	0.30889 (4)	0.13663 (3)	0.21159 (3)	0.03693 (12)	
O1	−0.05178 (12)	0.36372 (10)	0.34437 (10)	0.0491 (3)	
O2	0.15077 (17)	0.58243 (10)	0.29000 (11)	0.0647 (4)	
O3	0.2116 (2)	0.32476 (19)	−0.07349 (11)	0.1024 (6)	
O4	0.01130 (15)	0.28291 (12)	0.05809 (10)	0.0580 (3)	
N1	0.18901 (15)	0.47295 (12)	0.47309 (12)	0.0449 (3)	
H1N	0.145 (2)	0.5301 (17)	0.5141 (16)	0.054*	
N2	0.42620 (15)	0.38340 (12)	0.21313 (10)	0.0452 (3)	
C1	−0.11000 (18)	0.09770 (16)	0.37801 (14)	0.0466 (4)	
H1	−0.1955	0.1524	0.4109	0.056*	
C2	−0.1138 (2)	−0.02952 (17)	0.38248 (16)	0.0569 (4)	
H2	−0.2027	−0.0613	0.4179	0.068*	
C3	0.0142 (2)	−0.11030 (16)	0.33441 (16)	0.0556 (4)	
H3	0.0100	−0.1962	0.3379	0.067*	
C4	0.14759 (19)	−0.06681 (14)	0.28153 (14)	0.0448 (4)	
H4	0.2334	−0.1225	0.2503	0.054*	
C5	0.15164 (16)	0.06238 (12)	0.27566 (11)	0.0337 (3)	
C6	0.02306 (15)	0.14358 (13)	0.32381 (11)	0.0343 (3)	
C7	0.04308 (15)	0.27552 (13)	0.30944 (11)	0.0333 (3)	
C8	0.20210 (16)	0.29422 (12)	0.24261 (11)	0.0326 (3)	
C9	0.1918 (2)	0.38436 (14)	0.12494 (12)	0.0447 (4)	
H9	0.1131	0.4595	0.1411	0.054*	
C10	0.3518 (2)	0.42508 (18)	0.10187 (14)	0.0581 (4)	
H10A	0.4142	0.3851	0.0355	0.070*	
H10B	0.3404	0.5165	0.0835	0.070*	
C11	0.30303 (16)	0.36257 (12)	0.30895 (12)	0.0339 (3)	
C12	0.20227 (18)	0.48789 (13)	0.35267 (13)	0.0425 (3)	
C13	0.28113 (15)	0.36078 (13)	0.51820 (12)	0.0354 (3)	
C14	0.35773 (15)	0.29409 (12)	0.42447 (11)	0.0314 (3)	
C15	0.46804 (16)	0.18729 (13)	0.44844 (13)	0.0382 (3)	
H15	0.5232	0.1431	0.3867	0.046*	
C16	0.49556 (18)	0.14672 (15)	0.56609 (14)	0.0480 (4)	
H16	0.5709	0.0755	0.5832	0.058*	
C17	0.4122 (2)	0.21104 (16)	0.65801 (14)	0.0504 (4)	
H17	0.4297	0.1805	0.7365	0.060*	
C18	0.30339 (18)	0.31964 (15)	0.63592 (13)	0.0448 (4)	
H18	0.2475	0.3632	0.6978	0.054*	
C19	0.5353 (2)	0.4616 (2)	0.23767 (18)	0.0693 (5)	
H19A	0.6191	0.4613	0.1741	0.104*	
H19B	0.5777	0.4281	0.3116	0.104*	
H19C	0.4810	0.5470	0.2435	0.104*	
C20	0.1438 (2)	0.32731 (17)	0.02394 (14)	0.0562 (4)	
C21	−0.0480 (3)	0.2184 (2)	−0.02642 (17)	0.0750 (6)	
H21A	0.0326	0.1509	−0.0550	0.090*	
H21B	−0.0799	0.2776	−0.0944	0.090*	
C22	−0.1847 (4)	0.1653 (3)	0.0376 (2)	0.1083 (9)	
H22A	−0.2277	0.1220	−0.0158	0.162*	
H22B	−0.2633	0.2329	0.0658	0.162*	
H22C	−0.1514	0.1066	0.1043	0.162*	

### (I) Ethyl (2S*,2'R*)-1'-methyl-2'',3-dioxo-2,3-dihydrodispiro[1-benzothiophene-2,3'-pyrrolidine-2',3''-indoline]-4'-carboxylate Atomic displacement parameters (Å^2^)

**Table:**

	*U*^11^	*U*^22^	*U*^33^	*U*^12^	*U*^13^	*U*^23^
S1	0.0377 (2)	0.0370 (2)	0.0362 (2)	−0.00533 (14)	0.00290 (14)	−0.01255 (14)
O1	0.0431 (6)	0.0461 (6)	0.0584 (7)	0.0027 (5)	−0.0034 (5)	−0.0240 (5)
O2	0.0975 (10)	0.0318 (6)	0.0652 (8)	0.0029 (6)	−0.0309 (7)	−0.0053 (5)
O3	0.1453 (15)	0.1480 (16)	0.0305 (7)	−0.0721 (13)	0.0019 (8)	−0.0137 (8)
O4	0.0749 (8)	0.0634 (7)	0.0427 (6)	−0.0156 (6)	−0.0209 (6)	−0.0118 (5)
N1	0.0507 (7)	0.0394 (7)	0.0452 (7)	0.0031 (6)	−0.0083 (6)	−0.0197 (6)
N2	0.0532 (7)	0.0504 (7)	0.0361 (6)	−0.0256 (6)	0.0020 (5)	−0.0023 (5)
C1	0.0385 (8)	0.0581 (10)	0.0459 (8)	−0.0140 (7)	−0.0024 (6)	−0.0097 (7)
C2	0.0565 (10)	0.0616 (11)	0.0597 (10)	−0.0318 (9)	−0.0057 (8)	−0.0026 (8)
C3	0.0719 (11)	0.0419 (9)	0.0602 (10)	−0.0243 (8)	−0.0173 (9)	−0.0009 (7)
C4	0.0539 (9)	0.0347 (7)	0.0480 (8)	−0.0051 (6)	−0.0135 (7)	−0.0084 (6)
C5	0.0384 (7)	0.0344 (7)	0.0300 (6)	−0.0060 (5)	−0.0083 (5)	−0.0056 (5)
C6	0.0357 (7)	0.0381 (7)	0.0311 (7)	−0.0073 (6)	−0.0063 (5)	−0.0065 (5)
C7	0.0354 (7)	0.0380 (7)	0.0278 (6)	−0.0023 (6)	−0.0077 (5)	−0.0099 (5)
C8	0.0410 (7)	0.0302 (6)	0.0269 (6)	−0.0054 (5)	−0.0041 (5)	−0.0052 (5)
C9	0.0641 (10)	0.0407 (8)	0.0305 (7)	−0.0127 (7)	−0.0096 (6)	0.0015 (6)
C10	0.0809 (12)	0.0603 (10)	0.0365 (8)	−0.0313 (9)	−0.0019 (8)	0.0055 (7)
C11	0.0410 (7)	0.0304 (7)	0.0317 (7)	−0.0092 (5)	−0.0032 (5)	−0.0050 (5)
C12	0.0529 (9)	0.0322 (7)	0.0456 (8)	−0.0063 (6)	−0.0137 (7)	−0.0091 (6)
C13	0.0343 (7)	0.0383 (7)	0.0364 (7)	−0.0090 (6)	−0.0046 (5)	−0.0098 (6)
C14	0.0312 (6)	0.0332 (7)	0.0322 (7)	−0.0106 (5)	−0.0029 (5)	−0.0056 (5)
C15	0.0329 (7)	0.0383 (7)	0.0448 (8)	−0.0060 (6)	−0.0057 (6)	−0.0088 (6)
C16	0.0470 (8)	0.0441 (8)	0.0553 (9)	−0.0072 (7)	−0.0215 (7)	0.0000 (7)
C17	0.0611 (10)	0.0588 (10)	0.0368 (8)	−0.0214 (8)	−0.0183 (7)	0.0030 (7)
C18	0.0485 (8)	0.0576 (9)	0.0334 (7)	−0.0176 (7)	−0.0034 (6)	−0.0126 (6)
C19	0.0765 (13)	0.0797 (13)	0.0629 (11)	−0.0506 (11)	−0.0017 (9)	−0.0009 (10)
C20	0.0842 (13)	0.0554 (10)	0.0313 (8)	−0.0168 (9)	−0.0142 (8)	0.0018 (7)
C21	0.1095 (17)	0.0731 (13)	0.0542 (11)	−0.0218 (12)	−0.0389 (11)	−0.0129 (9)
C22	0.134 (2)	0.125 (2)	0.0921 (18)	−0.0653 (19)	−0.0405 (17)	−0.0196 (16)

### (I) Ethyl (2S*,2'R*)-1'-methyl-2'',3-dioxo-2,3-dihydrodispiro[1-benzothiophene-2,3'-pyrrolidine-2',3''-indoline]-4'-carboxylate Geometric parameters (Å, º)

**Table:**

S1—C5	1.7496 (14)	C9—C20	1.505 (2)
S1—C8	1.8330 (13)	C9—C10	1.522 (2)
O1—C7	1.2109 (16)	C9—H9	0.9800
O2—C12	1.2125 (18)	C10—H10A	0.9700
O3—C20	1.187 (2)	C10—H10B	0.9700
O4—C20	1.327 (2)	C11—C14	1.5063 (18)
O4—C21	1.450 (2)	C11—C12	1.5696 (19)
N1—C12	1.348 (2)	C13—C18	1.378 (2)
N1—C13	1.3972 (19)	C13—C14	1.3904 (18)
N1—H1N	0.827 (18)	C14—C15	1.3773 (19)
N2—C11	1.4561 (17)	C15—C16	1.387 (2)
N2—C19	1.457 (2)	C15—H15	0.9300
N2—C10	1.473 (2)	C16—C17	1.380 (2)
C1—C2	1.373 (2)	C16—H16	0.9300
C1—C6	1.388 (2)	C17—C18	1.380 (2)
C1—H1	0.9300	C17—H17	0.9300
C2—C3	1.381 (3)	C18—H18	0.9300
C2—H2	0.9300	C19—H19A	0.9600
C3—C4	1.375 (2)	C19—H19B	0.9600
C3—H3	0.9300	C19—H19C	0.9600
C4—C5	1.394 (2)	C21—C22	1.486 (3)
C4—H4	0.9300	C21—H21A	0.9700
C5—C6	1.3871 (19)	C21—H21B	0.9700
C6—C7	1.4523 (19)	C22—H22A	0.9600
C7—C8	1.5295 (18)	C22—H22B	0.9600
C8—C9	1.5495 (18)	C22—H22C	0.9600
C8—C11	1.5611 (18)		
			
C5—S1—C8	92.66 (6)	N2—C11—C8	99.56 (10)
C20—O4—C21	117.69 (15)	C14—C11—C8	116.51 (10)
C12—N1—C13	112.19 (12)	N2—C11—C12	114.06 (11)
C12—N1—H1N	122.9 (12)	C14—C11—C12	101.21 (10)
C13—N1—H1N	124.0 (12)	C8—C11—C12	110.24 (11)
C11—N2—C19	115.58 (13)	O2—C12—N1	126.23 (14)
C11—N2—C10	107.91 (12)	O2—C12—C11	126.46 (14)
C19—N2—C10	115.00 (13)	N1—C12—C11	107.26 (12)
C2—C1—C6	119.16 (15)	C18—C13—C14	122.52 (13)
C2—C1—H1	120.4	C18—C13—N1	127.67 (13)
C6—C1—H1	120.4	C14—C13—N1	109.76 (12)
C1—C2—C3	120.05 (15)	C15—C14—C13	119.22 (12)
C1—C2—H2	120.0	C15—C14—C11	131.91 (12)
C3—C2—H2	120.0	C13—C14—C11	108.80 (11)
C4—C3—C2	121.62 (15)	C14—C15—C16	118.95 (14)
C4—C3—H3	119.2	C14—C15—H15	120.5
C2—C3—H3	119.2	C16—C15—H15	120.5
C3—C4—C5	118.61 (15)	C17—C16—C15	120.63 (14)
C3—C4—H4	120.7	C17—C16—H16	119.7
C5—C4—H4	120.7	C15—C16—H16	119.7
C6—C5—C4	119.78 (13)	C18—C17—C16	121.38 (14)
C6—C5—S1	114.47 (10)	C18—C17—H17	119.3
C4—C5—S1	125.75 (11)	C16—C17—H17	119.3
C5—C6—C1	120.77 (13)	C13—C18—C17	117.16 (14)
C5—C6—C7	113.66 (12)	C13—C18—H18	121.4
C1—C6—C7	125.57 (13)	C17—C18—H18	121.4
O1—C7—C6	126.02 (13)	N2—C19—H19A	109.5
O1—C7—C8	121.74 (12)	N2—C19—H19B	109.5
C6—C7—C8	112.24 (11)	H19A—C19—H19B	109.5
C7—C8—C9	114.43 (11)	N2—C19—H19C	109.5
C7—C8—C11	115.22 (10)	H19A—C19—H19C	109.5
C9—C8—C11	99.72 (10)	H19B—C19—H19C	109.5
C7—C8—S1	106.91 (9)	O3—C20—O4	124.34 (17)
C9—C8—S1	110.04 (9)	O3—C20—C9	125.41 (18)
C11—C8—S1	110.43 (9)	O4—C20—C9	110.23 (14)
C20—C9—C10	115.35 (14)	O4—C21—C22	107.01 (17)
C20—C9—C8	114.02 (12)	O4—C21—H21A	110.3
C10—C9—C8	103.71 (12)	C22—C21—H21A	110.3
C20—C9—H9	107.8	O4—C21—H21B	110.3
C10—C9—H9	107.8	C22—C21—H21B	110.3
C8—C9—H9	107.8	H21A—C21—H21B	108.6
N2—C10—C9	105.51 (12)	C21—C22—H22A	109.5
N2—C10—H10A	110.6	C21—C22—H22B	109.5
C9—C10—H10A	110.6	H22A—C22—H22B	109.5
N2—C10—H10B	110.6	C21—C22—H22C	109.5
C9—C10—H10B	110.6	H22A—C22—H22C	109.5
H10A—C10—H10B	108.8	H22B—C22—H22C	109.5
N2—C11—C14	115.81 (11)		
			
C6—C1—C2—C3	0.6 (2)	C9—C8—C11—N2	−47.31 (12)
C1—C2—C3—C4	0.1 (3)	S1—C8—C11—N2	68.47 (11)
C2—C3—C4—C5	−0.7 (2)	C7—C8—C11—C14	64.43 (15)
C3—C4—C5—C6	0.6 (2)	C9—C8—C11—C14	−172.58 (11)
C3—C4—C5—S1	−179.58 (11)	S1—C8—C11—C14	−56.80 (13)
C8—S1—C5—C6	−2.05 (11)	C7—C8—C11—C12	−50.11 (14)
C8—S1—C5—C4	178.17 (12)	C9—C8—C11—C12	72.88 (13)
C4—C5—C6—C1	0.0 (2)	S1—C8—C11—C12	−171.35 (9)
S1—C5—C6—C1	−179.75 (11)	C13—N1—C12—O2	−170.75 (15)
C4—C5—C6—C7	−179.06 (12)	C13—N1—C12—C11	6.75 (16)
S1—C5—C6—C7	1.15 (15)	N2—C11—C12—O2	43.7 (2)
C2—C1—C6—C5	−0.7 (2)	C14—C11—C12—O2	168.82 (15)
C2—C1—C6—C7	178.32 (14)	C8—C11—C12—O2	−67.27 (19)
C5—C6—C7—O1	−179.54 (13)	N2—C11—C12—N1	−133.76 (13)
C1—C6—C7—O1	1.4 (2)	C14—C11—C12—N1	−8.68 (14)
C5—C6—C7—C8	0.68 (16)	C8—C11—C12—N1	115.23 (13)
C1—C6—C7—C8	−178.36 (13)	C12—N1—C13—C18	175.59 (14)
O1—C7—C8—C9	−59.73 (17)	C12—N1—C13—C14	−1.69 (17)
C6—C7—C8—C9	120.06 (12)	C18—C13—C14—C15	−4.4 (2)
O1—C7—C8—C11	55.04 (17)	N1—C13—C14—C15	173.00 (12)
C6—C7—C8—C11	−125.17 (12)	C18—C13—C14—C11	178.13 (12)
O1—C7—C8—S1	178.16 (11)	N1—C13—C14—C11	−4.43 (15)
C6—C7—C8—S1	−2.05 (12)	N2—C11—C14—C15	−45.34 (19)
C5—S1—C8—C7	2.25 (9)	C8—C11—C14—C15	71.24 (18)
C5—S1—C8—C9	−122.58 (10)	C12—C11—C14—C15	−169.23 (14)
C5—S1—C8—C11	128.30 (9)	N2—C11—C14—C13	131.64 (12)
C7—C8—C9—C20	−73.99 (17)	C8—C11—C14—C13	−111.78 (12)
C11—C8—C9—C20	162.46 (14)	C12—C11—C14—C13	7.76 (13)
S1—C8—C9—C20	46.39 (16)	C13—C14—C15—C16	2.27 (19)
C7—C8—C9—C10	159.77 (12)	C11—C14—C15—C16	178.99 (13)
C11—C8—C9—C10	36.22 (14)	C14—C15—C16—C17	1.0 (2)
S1—C8—C9—C10	−79.85 (13)	C15—C16—C17—C18	−2.3 (2)
C11—N2—C10—C9	−19.43 (17)	C14—C13—C18—C17	3.1 (2)
C19—N2—C10—C9	−150.08 (15)	N1—C13—C18—C17	−173.84 (14)
C20—C9—C10—N2	−137.43 (14)	C16—C17—C18—C13	0.3 (2)
C8—C9—C10—N2	−12.03 (16)	C21—O4—C20—O3	4.9 (3)
C19—N2—C11—C14	−61.82 (18)	C21—O4—C20—C9	−176.82 (15)
C10—N2—C11—C14	167.85 (12)	C10—C9—C20—O3	−8.5 (3)
C19—N2—C11—C8	172.43 (14)	C8—C9—C20—O3	−128.4 (2)
C10—N2—C11—C8	42.11 (14)	C10—C9—C20—O4	173.27 (14)
C19—N2—C11—C12	55.08 (18)	C8—C9—C20—O4	53.38 (19)
C10—N2—C11—C12	−75.25 (15)	C20—O4—C21—C22	173.44 (19)
C7—C8—C11—N2	−170.30 (11)		

### (I) Ethyl (2S*,2'R*)-1'-methyl-2'',3-dioxo-2,3-dihydrodispiro[1-benzothiophene-2,3'-pyrrolidine-2',3''-indoline]-4'-carboxylate Hydrogen-bond geometry (Å, º)

Cg is the centroid of the C1–C6 ring.

**Table:**

*D*—H···*A*	*D*—H	H···*A*	*D*···*A*	*D*—H···*A*
N1—H1*N*···O1^i^	0.83 (2)	2.09 (2)	2.890 (2)	164 (2)
C3—H3···O2^ii^	0.93	2.56	3.385 (2)	148
C18—H18···O3^iii^	0.93	2.56	3.299 (2)	136
C21—H21*B*···O2^iv^	0.97	2.59	3.560 (2)	174
C2—H2···*Cg*^v^	0.93	2.81	3.649 (2)	151

Symmetry codes: (i) −*x*, −*y*+1, −*z*+1; (ii) *x*, *y*−1, *z*; (iii) *x*, *y*, *z*+1; (iv) −*x*, −*y*+1, −*z*; (v) −*x*, −*y*, −*z*+1.

### (II) Ethyl (2S*,2'R*)-5''-chloro-1'-methyl-2'',3-dioxo-2,3-dihydrodispiro[1-benzothiophene-2,3'-pyrrolidine-2',3''-indoline]-4'-carboxylate Crystal data

**Table:**

C_22_H_19_ClN_2_O_4_S	*V* = 1050.26 (9) Å^3^
*M**_r_* = 442.90	*Z* = 2
Triclinic, *P*1	*F*(000) = 460
Hall symbol: -P 1	*D*_x_ = 1.401 Mg m^−^^3^
*a* = 10.4678 (5) Å	Mo *K*α radiation, λ = 0.71073 Å
*b* = 10.9074 (5) Å	θ = 2.0–25.0°
*c* = 11.5652 (5) Å	µ = 0.31 mm^−^^1^
α = 85.973 (2)°	*T* = 293 K
β = 65.612 (2)°	Block, colourless
γ = 62.089 (2)°	0.35 × 0.30 × 0.30 mm

### (II) Ethyl (2S*,2'R*)-5''-chloro-1'-methyl-2'',3-dioxo-2,3-dihydrodispiro[1-benzothiophene-2,3'-pyrrolidine-2',3''-indoline]-4'-carboxylate Data collection

**Table:**

Bruker AXS kappa APEX2 CCD diffractometer	*R*_int_ = 0.024
Absorption correction: multi-scan (*SADABS*; Sheldrick, 1996)	θ_max_ = 25.3°, θ_min_ = 2.1°
*T*_min_ = 0.931, *T*_max_ = 0.940	*h* = −12→10
16876 measured reflections	*k* = −13→11
3788 independent reflections	*l* = −13→12
3178 reflections with *I* > 2σ(*I*)	

### (II) Ethyl (2S*,2'R*)-5''-chloro-1'-methyl-2'',3-dioxo-2,3-dihydrodispiro[1-benzothiophene-2,3'-pyrrolidine-2',3''-indoline]-4'-carboxylate Refinement

**Table:**

Refinement on *F*^2^	Primary atom site location: structure-invariant direct methods
Least-squares matrix: full	Secondary atom site location: difference Fourier map
*R*[*F*^2^ > 2σ(*F*^2^)] = 0.036	Hydrogen site location: mixed
*wR*(*F*^2^) = 0.103	H atoms treated by a mixture of independent and constrained refinement
*S* = 1.11	*w* = 1/[σ^2^(*F*_o_^2^) + (0.0458*P*)^2^ + 0.3849*P*] where *P* = (*F*_o_^2^ + 2*F*_c_^2^)/3
3788 reflections	(Δ/σ)_max_ < 0.001
276 parameters	Δρ_max_ = 0.29 e Å^−^^3^
0 restraints	Δρ_min_ = −0.27 e Å^−^^3^

### (II) Ethyl (2S*,2'R*)-5''-chloro-1'-methyl-2'',3-dioxo-2,3-dihydrodispiro[1-benzothiophene-2,3'-pyrrolidine-2',3''-indoline]-4'-carboxylate Special details

**Table:**

Geometry. All esds (except the esd in the dihedral angle between two l.s. planes) are estimated using the full covariance matrix. The cell esds are taken into account individually in the estimation of esds in distances, angles and torsion angles; correlations between esds in cell parameters are only used when they are defined by crystal symmetry. An approximate (isotropic) treatment of cell esds is used for estimating esds involving l.s. planes.

### (II) Ethyl (2S*,2'R*)-5''-chloro-1'-methyl-2'',3-dioxo-2,3-dihydrodispiro[1-benzothiophene-2,3'-pyrrolidine-2',3''-indoline]-4'-carboxylate Fractional atomic coordinates and isotropic or equivalent isotropic displacement parameters (Å^2^)

**Table:**

	*x*	*y*	*z*	*U*_iso_*/*U*_eq_	
Cl1	0.78453 (7)	−0.07384 (7)	0.27294 (5)	0.06115 (18)	
S1	0.47022 (6)	0.40379 (5)	0.61542 (4)	0.04426 (15)	
O1	0.24464 (18)	0.30234 (17)	0.93183 (14)	0.0622 (4)	
O2	0.5511 (2)	0.14387 (16)	0.96174 (13)	0.0596 (4)	
O3	0.5065 (3)	0.6187 (2)	0.8095 (3)	0.1135 (8)	
O4	0.2715 (3)	0.62165 (17)	0.89290 (19)	0.0794 (5)	
N1	0.5579 (2)	−0.00867 (18)	0.83019 (16)	0.0476 (4)	
H1N	0.534 (3)	−0.054 (2)	0.886 (2)	0.057*	
N2	0.7301 (2)	0.20503 (19)	0.70036 (16)	0.0493 (4)	
C1	0.0252 (3)	0.4697 (2)	0.8114 (2)	0.0612 (6)	
H1	−0.0287	0.4448	0.8874	0.073*	
C2	−0.0533 (3)	0.5469 (3)	0.7396 (3)	0.0782 (8)	
H2	−0.1613	0.5752	0.7672	0.094*	
C3	0.0283 (3)	0.5822 (3)	0.6265 (3)	0.0793 (8)	
H3	−0.0262	0.6340	0.5788	0.095*	
C4	0.1878 (3)	0.5431 (3)	0.5823 (2)	0.0629 (6)	
H4	0.2411	0.5676	0.5058	0.075*	
C5	0.2670 (2)	0.4661 (2)	0.65526 (19)	0.0444 (4)	
C6	0.1862 (2)	0.42945 (19)	0.76880 (18)	0.0443 (4)	
C7	0.2853 (2)	0.34936 (19)	0.83467 (17)	0.0420 (4)	
C8	0.4562 (2)	0.33081 (19)	0.76494 (16)	0.0378 (4)	
C9	0.4971 (3)	0.4035 (2)	0.84540 (19)	0.0498 (5)	
H9	0.4533	0.3873	0.9344	0.060*	
C10	0.6786 (3)	0.3230 (3)	0.7914 (2)	0.0643 (6)	
H10	0.7241	0.3823	0.7485	0.077*	
H11	0.7118	0.2898	0.8595	0.077*	
C11	0.5959 (2)	0.17760 (19)	0.73600 (16)	0.0378 (4)	
C12	0.5630 (2)	0.1056 (2)	0.85934 (18)	0.0448 (4)	
C13	0.6002 (2)	−0.03474 (19)	0.69931 (17)	0.0396 (4)	
C14	0.6242 (2)	0.07240 (18)	0.63836 (16)	0.0358 (4)	
C15	0.6818 (2)	0.0617 (2)	0.50663 (17)	0.0394 (4)	
H15	0.7009	0.1311	0.4639	0.047*	
C16	0.7104 (2)	−0.0557 (2)	0.43996 (18)	0.0439 (4)	
C17	0.6825 (2)	−0.1597 (2)	0.5010 (2)	0.0491 (5)	
H17	0.7014	−0.2364	0.4533	0.059*	
C18	0.6265 (2)	−0.1503 (2)	0.6330 (2)	0.0492 (5)	
H18	0.6074	−0.2198	0.6755	0.059*	
C19	0.8815 (3)	0.0837 (3)	0.6825 (3)	0.0811 (8)	
H22A	0.9067	0.0101	0.6227	0.122*	
H22B	0.8732	0.0515	0.7634	0.122*	
H19	0.9647	0.1094	0.6496	0.122*	
C20	0.4289 (4)	0.5589 (3)	0.8460 (3)	0.0688 (7)	
C21	0.1907 (6)	0.7722 (3)	0.8925 (5)	0.1379 (18)	
H20A	0.1867	0.8208	0.9620	0.165*	
H20B	0.2502	0.7944	0.8122	0.165*	
C22	0.0345 (5)	0.8180 (3)	0.9074 (5)	0.1294 (15)	
H21A	−0.0217	0.9186	0.9177	0.194*	
H21B	−0.0205	0.7869	0.9820	0.194*	
H21C	0.0389	0.7797	0.8328	0.194*	

### (II) Ethyl (2S*,2'R*)-5''-chloro-1'-methyl-2'',3-dioxo-2,3-dihydrodispiro[1-benzothiophene-2,3'-pyrrolidine-2',3''-indoline]-4'-carboxylate Atomic displacement parameters (Å^2^)

**Table:**

	*U*^11^	*U*^22^	*U*^33^	*U*^12^	*U*^13^	*U*^23^
Cl1	0.0577 (3)	0.0831 (4)	0.0385 (3)	−0.0329 (3)	−0.0163 (2)	−0.0048 (2)
S1	0.0478 (3)	0.0544 (3)	0.0399 (3)	−0.0309 (2)	−0.0221 (2)	0.0220 (2)
O1	0.0585 (9)	0.0682 (10)	0.0430 (8)	−0.0321 (8)	−0.0069 (7)	0.0171 (7)
O2	0.0964 (12)	0.0631 (9)	0.0399 (8)	−0.0478 (9)	−0.0388 (8)	0.0224 (7)
O3	0.158 (2)	0.0845 (14)	0.163 (2)	−0.0951 (16)	−0.0874 (18)	0.0504 (14)
O4	0.1093 (16)	0.0397 (9)	0.0970 (14)	−0.0343 (10)	−0.0535 (12)	0.0116 (8)
N1	0.0641 (11)	0.0461 (9)	0.0387 (9)	−0.0316 (8)	−0.0234 (8)	0.0172 (7)
N2	0.0486 (9)	0.0683 (11)	0.0483 (9)	−0.0363 (9)	−0.0274 (8)	0.0151 (8)
C1	0.0441 (11)	0.0570 (13)	0.0658 (14)	−0.0223 (10)	−0.0099 (10)	−0.0021 (11)
C2	0.0431 (12)	0.0802 (18)	0.101 (2)	−0.0206 (12)	−0.0317 (14)	0.0097 (16)
C3	0.0645 (16)	0.0875 (19)	0.095 (2)	−0.0303 (14)	−0.0514 (16)	0.0261 (16)
C4	0.0618 (14)	0.0712 (15)	0.0665 (14)	−0.0327 (12)	−0.0387 (12)	0.0247 (12)
C5	0.0468 (11)	0.0438 (10)	0.0456 (11)	−0.0231 (9)	−0.0211 (9)	0.0070 (8)
C6	0.0425 (10)	0.0387 (10)	0.0453 (11)	−0.0183 (8)	−0.0138 (9)	−0.0021 (8)
C7	0.0460 (10)	0.0368 (10)	0.0355 (10)	−0.0215 (8)	−0.0084 (8)	0.0001 (8)
C8	0.0491 (10)	0.0410 (10)	0.0314 (9)	−0.0281 (8)	−0.0180 (8)	0.0115 (7)
C9	0.0785 (14)	0.0518 (12)	0.0426 (11)	−0.0435 (11)	−0.0336 (10)	0.0153 (9)
C10	0.0840 (17)	0.0814 (16)	0.0663 (15)	−0.0578 (14)	−0.0467 (13)	0.0207 (12)
C11	0.0434 (10)	0.0463 (10)	0.0322 (9)	−0.0260 (8)	−0.0197 (8)	0.0136 (7)
C12	0.0554 (11)	0.0462 (11)	0.0364 (10)	−0.0252 (9)	−0.0234 (9)	0.0144 (8)
C13	0.0366 (9)	0.0421 (10)	0.0389 (10)	−0.0175 (8)	−0.0174 (8)	0.0087 (8)
C14	0.0322 (8)	0.0405 (9)	0.0348 (9)	−0.0167 (7)	−0.0159 (7)	0.0069 (7)
C15	0.0341 (9)	0.0495 (11)	0.0367 (9)	−0.0211 (8)	−0.0163 (8)	0.0084 (8)
C16	0.0334 (9)	0.0574 (12)	0.0380 (10)	−0.0188 (9)	−0.0153 (8)	0.0004 (8)
C17	0.0448 (11)	0.0481 (11)	0.0536 (12)	−0.0193 (9)	−0.0226 (9)	−0.0035 (9)
C18	0.0494 (11)	0.0431 (11)	0.0589 (13)	−0.0238 (9)	−0.0254 (10)	0.0100 (9)
C19	0.0530 (14)	0.104 (2)	0.091 (2)	−0.0317 (14)	−0.0412 (14)	0.0100 (16)
C20	0.116 (2)	0.0585 (14)	0.0698 (16)	−0.0582 (16)	−0.0565 (16)	0.0242 (12)
C21	0.190 (5)	0.0420 (16)	0.224 (5)	−0.044 (2)	−0.142 (4)	0.033 (2)
C22	0.142 (4)	0.060 (2)	0.162 (4)	−0.028 (2)	−0.071 (3)	0.034 (2)

### (II) Ethyl (2S*,2'R*)-5''-chloro-1'-methyl-2'',3-dioxo-2,3-dihydrodispiro[1-benzothiophene-2,3'-pyrrolidine-2',3''-indoline]-4'-carboxylate Geometric parameters (Å, º)

**Table:**

Cl1—C16	1.7448 (19)	C8—C11	1.559 (3)
S1—C5	1.755 (2)	C9—C20	1.499 (3)
S1—C8	1.8308 (17)	C9—C10	1.519 (3)
O1—C7	1.198 (2)	C9—H9	0.9800
O2—C12	1.221 (2)	C10—H10	0.9700
O3—C20	1.199 (3)	C10—H11	0.9700
O4—C20	1.318 (3)	C11—C14	1.506 (2)
O4—C21	1.454 (3)	C11—C12	1.568 (2)
N1—C12	1.344 (3)	C13—C18	1.374 (3)
N1—C13	1.399 (2)	C13—C14	1.392 (2)
N1—H1N	0.81 (2)	C14—C15	1.378 (2)
N2—C19	1.453 (3)	C15—C16	1.384 (3)
N2—C10	1.459 (3)	C15—H15	0.9300
N2—C11	1.460 (2)	C16—C17	1.377 (3)
C1—C2	1.376 (4)	C17—C18	1.382 (3)
C1—C6	1.388 (3)	C17—H17	0.9300
C1—H1	0.9300	C18—H18	0.9300
C2—C3	1.379 (4)	C19—H22A	0.9600
C2—H2	0.9300	C19—H22B	0.9600
C3—C4	1.374 (3)	C19—H19	0.9600
C3—H3	0.9300	C21—C22	1.402 (6)
C4—C5	1.390 (3)	C21—H20A	0.9700
C4—H4	0.9300	C21—H20B	0.9700
C5—C6	1.388 (3)	C22—H21A	0.9600
C6—C7	1.460 (3)	C22—H21B	0.9600
C7—C8	1.543 (3)	C22—H21C	0.9600
C8—C9	1.558 (3)		
			
C5—S1—C8	93.13 (9)	N2—C11—C8	100.04 (14)
C20—O4—C21	116.5 (3)	C14—C11—C8	119.28 (14)
C12—N1—C13	111.90 (16)	N2—C11—C12	114.02 (14)
C12—N1—H1N	120.4 (16)	C14—C11—C12	101.08 (14)
C13—N1—H1N	127.7 (16)	C8—C11—C12	109.68 (14)
C19—N2—C10	114.16 (18)	O2—C12—N1	126.35 (17)
C19—N2—C11	115.48 (18)	O2—C12—C11	125.72 (17)
C10—N2—C11	107.82 (16)	N1—C12—C11	107.87 (15)
C2—C1—C6	119.0 (2)	C18—C13—C14	122.24 (17)
C2—C1—H1	120.5	C18—C13—N1	127.88 (17)
C6—C1—H1	120.5	C14—C13—N1	109.75 (16)
C1—C2—C3	119.9 (2)	C15—C14—C13	119.77 (17)
C1—C2—H2	120.0	C15—C14—C11	130.86 (16)
C3—C2—H2	120.0	C13—C14—C11	109.00 (15)
C4—C3—C2	122.0 (2)	C14—C15—C16	117.82 (17)
C4—C3—H3	119.0	C14—C15—H15	121.1
C2—C3—H3	119.0	C16—C15—H15	121.1
C3—C4—C5	118.1 (2)	C17—C16—C15	122.18 (18)
C3—C4—H4	120.9	C17—C16—Cl1	118.56 (15)
C5—C4—H4	120.9	C15—C16—Cl1	119.27 (15)
C6—C5—C4	120.30 (19)	C16—C17—C18	120.25 (19)
C6—C5—S1	114.19 (14)	C16—C17—H17	119.9
C4—C5—S1	125.49 (17)	C18—C17—H17	119.9
C5—C6—C1	120.6 (2)	C13—C18—C17	117.71 (18)
C5—C6—C7	113.80 (17)	C13—C18—H18	121.1
C1—C6—C7	125.61 (19)	C17—C18—H18	121.1
O1—C7—C6	126.40 (18)	N2—C19—H22A	109.5
O1—C7—C8	121.59 (18)	N2—C19—H22B	109.5
C6—C7—C8	112.01 (15)	H22A—C19—H22B	109.5
C7—C8—C9	113.63 (15)	N2—C19—H19	109.5
C7—C8—C11	115.91 (14)	H22A—C19—H19	109.5
C9—C8—C11	100.13 (14)	H22B—C19—H19	109.5
C7—C8—S1	106.50 (12)	O3—C20—O4	124.3 (3)
C9—C8—S1	110.40 (12)	O3—C20—C9	124.8 (3)
C11—C8—S1	110.23 (12)	O4—C20—C9	110.9 (2)
C20—C9—C10	114.2 (2)	C22—C21—O4	110.4 (3)
C20—C9—C8	114.23 (17)	C22—C21—H20A	109.6
C10—C9—C8	104.53 (16)	O4—C21—H20A	109.6
C20—C9—H9	107.9	C22—C21—H20B	109.6
C10—C9—H9	107.9	O4—C21—H20B	109.6
C8—C9—H9	107.9	H20A—C21—H20B	108.1
N2—C10—C9	105.75 (16)	C21—C22—H21A	109.5
N2—C10—H10	110.6	C21—C22—H21B	109.5
C9—C10—H10	110.6	H21A—C22—H21B	109.5
N2—C10—H11	110.6	C21—C22—H21C	109.5
C9—C10—H11	110.6	H21A—C22—H21C	109.5
H10—C10—H11	108.7	H21B—C22—H21C	109.5
N2—C11—C14	113.34 (15)		
			
C6—C1—C2—C3	0.5 (4)	S1—C8—C11—N2	71.69 (14)
C1—C2—C3—C4	−0.3 (5)	C7—C8—C11—C14	68.6 (2)
C2—C3—C4—C5	−0.2 (4)	C9—C8—C11—C14	−168.71 (15)
C3—C4—C5—C6	0.5 (3)	S1—C8—C11—C14	−52.40 (18)
C3—C4—C5—S1	179.1 (2)	C7—C8—C11—C12	−47.1 (2)
C8—S1—C5—C6	−4.41 (16)	C9—C8—C11—C12	75.54 (16)
C8—S1—C5—C4	177.0 (2)	S1—C8—C11—C12	−168.15 (12)
C4—C5—C6—C1	−0.3 (3)	C13—N1—C12—O2	−170.6 (2)
S1—C5—C6—C1	−179.02 (16)	C13—N1—C12—C11	6.5 (2)
C4—C5—C6—C7	−179.70 (19)	N2—C11—C12—O2	49.3 (3)
S1—C5—C6—C7	1.6 (2)	C14—C11—C12—O2	171.21 (19)
C2—C1—C6—C5	−0.2 (3)	C8—C11—C12—O2	−62.0 (2)
C2—C1—C6—C7	179.1 (2)	N2—C11—C12—N1	−127.93 (18)
C5—C6—C7—O1	−177.65 (19)	C14—C11—C12—N1	−5.98 (19)
C1—C6—C7—O1	3.0 (3)	C8—C11—C12—N1	120.84 (17)
C5—C6—C7—C8	2.9 (2)	C12—N1—C13—C18	171.55 (19)
C1—C6—C7—C8	−176.43 (18)	C12—N1—C13—C14	−4.3 (2)
O1—C7—C8—C9	−63.4 (2)	C18—C13—C14—C15	−2.4 (3)
C6—C7—C8—C9	116.04 (17)	N1—C13—C14—C15	173.75 (15)
O1—C7—C8—C11	51.8 (2)	C18—C13—C14—C11	−176.13 (16)
C6—C7—C8—C11	−128.75 (16)	N1—C13—C14—C11	0.0 (2)
O1—C7—C8—S1	174.81 (16)	N2—C11—C14—C15	−46.9 (2)
C6—C7—C8—S1	−5.73 (18)	C8—C11—C14—C15	70.5 (2)
C5—S1—C8—C7	5.57 (13)	C12—C11—C14—C15	−169.32 (18)
C5—S1—C8—C9	−118.22 (15)	N2—C11—C14—C13	125.89 (16)
C5—S1—C8—C11	132.08 (13)	C8—C11—C14—C13	−116.74 (16)
C7—C8—C9—C20	−78.9 (2)	C12—C11—C14—C13	3.47 (18)
C11—C8—C9—C20	156.84 (19)	C13—C14—C15—C16	1.4 (2)
S1—C8—C9—C20	40.7 (2)	C11—C14—C15—C16	173.56 (17)
C7—C8—C9—C10	155.62 (16)	C14—C15—C16—C17	0.3 (3)
C11—C8—C9—C10	31.38 (17)	C14—C15—C16—Cl1	−179.61 (13)
S1—C8—C9—C10	−84.80 (16)	C15—C16—C17—C18	−1.0 (3)
C19—N2—C10—C9	−153.17 (19)	Cl1—C16—C17—C18	178.82 (14)
C11—N2—C10—C9	−23.4 (2)	C14—C13—C18—C17	1.6 (3)
C20—C9—C10—N2	−132.10 (19)	N1—C13—C18—C17	−173.82 (18)
C8—C9—C10—N2	−6.6 (2)	C16—C17—C18—C13	0.1 (3)
C19—N2—C11—C14	−59.7 (2)	C21—O4—C20—O3	3.5 (4)
C10—N2—C11—C14	171.34 (16)	C21—O4—C20—C9	−176.8 (3)
C19—N2—C11—C8	172.22 (17)	C10—C9—C20—O3	−0.9 (4)
C10—N2—C11—C8	43.22 (18)	C8—C9—C20—O3	−121.1 (3)
C19—N2—C11—C12	55.3 (2)	C10—C9—C20—O4	179.43 (19)
C10—N2—C11—C12	−73.7 (2)	C8—C9—C20—O4	59.2 (3)
C7—C8—C11—N2	−167.27 (14)	C20—O4—C21—C22	162.3 (3)
C9—C8—C11—N2	−44.62 (16)		

### (II) Ethyl (2S*,2'R*)-5''-chloro-1'-methyl-2'',3-dioxo-2,3-dihydrodispiro[1-benzothiophene-2,3'-pyrrolidine-2',3''-indoline]-4'-carboxylate Hydrogen-bond geometry (Å, º)

Cg is the centroid of the C1–C6 ring.

**Table:**

*D*—H···*A*	*D*—H	H···*A*	*D*···*A*	*D*—H···*A*
N1—H1*N*···O2^i^	0.81 (2)	2.03 (2)	2.842 (2)	172 (2)
C18—H18···O3^ii^	0.93	2.57	3.496 (3)	171
C2—H2···*Cg*^iii^	0.93	2.83	3.649 (2)	155

Symmetry codes: (i) −*x*+1, −*y*, −*z*+2; (ii) *x*, *y*−1, *z*; (iii) −*x*, −*y*, −*z*+1.

## References

[bb1] Allen, F. H. (2002). *Acta Cryst.* B**58**, 380–388. 10.1107/s0108768102003890 12037359

[bb2] Bruker (2004). *APEX2*, *SAINT* and *XPREP*. Bruker AXS Inc., Madison, Wisconsin, USA.

[bb3] Cordell, G. (1981). In *Introduction to Alkaloids: A Biogenic Approach*. New York: Wiley International.

[bb4] Govind, M. M., Selvanayagam, S., Velmurugan, D., Ravikumar, K., Sridhar, G. & Raghunathan, R. (2003). *Acta Cryst.* E**59**, o1438–o1440.

[bb5] Huryn, D. M., Trost, B. M. & Fleming, I. (1991). *Comp. Org. Synth.* **1**, 64–74.

[bb6] Kannan, P. S., Lanka, S., Thennarasu, S., Vimala, G. & SubbiahPandi, A. (2013*a*). *Acta Cryst.* E**69**, o854–o855. 10.1107/S1600536813011537 PMC3684937 23795039

[bb7] Kannan, P. S., Yuvaraj, P. S., Manivannan, K., Reddy, B. S. R. & SubbiahPandi, A. (2013*b*). *Acta Cryst.* E**69**, o825–o826. 10.1107/S1600536813011501 PMC3684914 23795016

[bb8] Macrae, C. F., Bruno, I. J., Chisholm, J. A., Edgington, P. R., McCabe, P., Pidcock, E., Rodriguez-Monge, L., Taylor, R., van de Streek, J. & Wood, P. A. (2008). *J. Appl. Cryst.* **41**, 466–470.

[bb9] Sheldrick, G. M. (1996). *SADABS*. University of Göttingen, Germany.

[bb10] Sheldrick, G. M. (2008). *Acta Cryst.* A**64**, 112–122. 10.1107/S0108767307043930 18156677

[bb11] Spek, A. L. (2009). *Acta Cryst.* D**65**, 148–155. 10.1107/S090744490804362X PMC2631630 19171970

[bb12] Suzuki, H., Aoyagi, S. & Kibayashi, C. (1994). *Tetrahedron Lett.* **35**, 6119–6122.

[bb13] Waldmann, H. (1995). *Synlett*. pp. 133–141.

[bb14] Westrip, S. P. (2010). *J. Appl. Cryst.* **43**, 920–925.

